# Multidimensional Modification and Functional Optimization of Melittin: From Natural Toxic Peptide to Safe and Effective Therapeutics

**DOI:** 10.3390/pharmaceutics18070840

**Published:** 2026-07-09

**Authors:** Zhengyu Chen, Chunli Su, Tianyao Guo, Zhiting Fang, Yujie Peng, Xiantao Yang, Hongli Liao

**Affiliations:** 1 School of Pharmacy, Chengdu Medical College, 783, Xindu Avenue, Xindu District, Chengdu 610500, China; 17764973335@163.com (Z.C.); 13890975507@163.com (T.G.); 18980498963@163.com (Z.F.); 2 School of Public Health, Chengdu Medical College, 783 Xindu Avenue, Xindu District, Chengdu 610500, China; suchunli@cmc.edu.cn; 3 Yibin Center for Disease Control and Prevention, Yibin 644600, China; pengyujie0624@126.com

**Keywords:** melittin, peptide, structural modification

## Abstract

Melittin, a cationic amphipathic 26-residue peptide from bee venom, displays broad-spectrum antibacterial, antitumor and anti-inflammatory activities, yet severe hemolysis, poor cell selectivity, rapid plasma degradation and high immunogenicity hinder its clinical translation. Structural modification is a vital strategy to overcome its druggability limitations. This review systematically summarizes three mainstream modification approaches of melittin: sequence remodeling, chemical derivatization and conjugate engineering. We illustrate how these strategies tune melittin’s conformation, charge and amphiphilicity to lower toxicity and immunogenicity, improve in vivo stability, and enable targeted stimulus-responsive delivery, while unraveling its core functional sites and mechanisms. Current research gaps and future directions including combined modification, multifunctional intelligent conjugates, preclinical safety assessment and scaled production optimization are discussed, providing theoretical support for developing safe, effective melittin-based therapeutics.

## 1. Introduction

**Figure 1:**
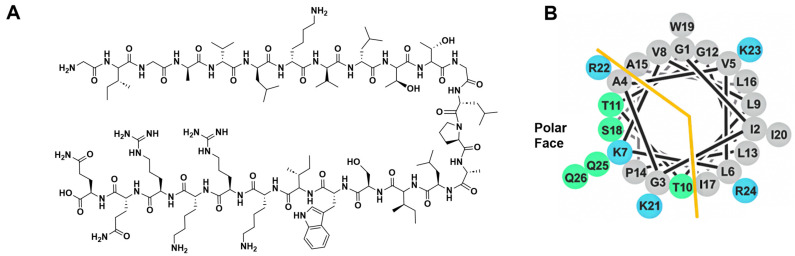
(**A**) Chemical structure of linear melittin polypeptide; (**B**) the helical wheel diagram of melittin showing the polar and non-polar surfaces of the helix (all residues were plotted in a helical conformation: the yellow line is the dividing line between the polar face and the non-polar face of the amphipathic alpha helix of melittin, the gray symbol represents hydrophobic residues, the green symbol represents polar and uncharged residues, and the blue symbol represents basic residues). Adapted from Reference [13].

Melittin is the core active peptide in the venom of honeybees (mainly Apis melitensis), accounting for 40% to 60% of the dry weight of bee venom, and can also be artificially prepared by solid-phase synthesis [1]. The primary structure is composed of 26 amino acid residues (GIGAVLKVLTTGLPALISWIKRKRQQ); the sequence is rich in lysine, arginine, and other positively charged amino acids, with typical cationic amphiphilic characteristics (Figure 1A). The structural characteristics lay the groundwork for its interaction with the cell membrane [2]. In the presence of neutral pH, trace concentration and secondary structure inducers such as trifluoroethanol (TFE), melittin could form a continuous helical structure consisting of an α-helix at the N terminus, an α-helix at the C terminus and a non-canonical 310-helix at the middle region. This special helical conformation is essential for its biological activity [3]. It is particularly crucial that its helical structure has a clear amphiphilic partition, and the reasonable arrangement of hydrophobic and hydrophilic regions enables it to quickly bind to the cell membrane and form pores (Figure 1B), causing the destruction of the cell membrane, thereby exerting antibacterial, anti-tumor activities and hemolytic toxicity [4]. Melittin exhibits strong aggregation propensity under physiological conditions, which is dominated by reversible tetramer formation. Tetrameric oligomerization drastically enhances membrane lysis toward zwitterionic red blood cell lipids, while anionic bacterial lipid bilayers suppress large oligomer assembly to preserve the peptide’s antibacterial activity [5].

Based on its unique structural features, melittin exhibits rich and potent pharmacological activities and has important potential for anti-infection, anti-tumor and anti-inflammatory therapy [4]. It can play a significant role in both in vitro and in vivo experiments, and has potential application value for diseases that lack specific therapeutic drugs. It can also synergise with antibiotics such as ciprofloxacin and rifampicin to improve the efficacy [6]. The clinical translation of melittin is limited by multiple inherent defects, with the central problems of toxicity and insufficient cell selectivity [7]. As it contains a large number of positively charged amino acids, it can penetrate the cell membrane non-specifically and cause hemolysis of erythrocytes and damage of normal cells. In vitro experiments showed that the median effective concentration (HD_50_) of hemolysis was only 0.44 µg/mL, and the median effective concentration (IC_50_) of cytotoxicity was 6.45 µg/mL. The median lethal dose (LD_50_) of intraperitoneal injection in BALB/c mice was up to 4.98 mg/kg, and the antibacterial effect was not effective at a safe sublethal dose (2.4 mg/kg), forming a contradiction between efficacy and toxicity [8]. At the same time, its poor pharmacokinetic properties, rapid degradation in plasma, low bioavailability, immunogenicity and possible allergic reactions, coupled with the high cost of large-scale preparation, further hinder its clinical application [9]. 

Despite these limitations, melittin has multiple advantages over other insect-derived antimicrobial peptides such as cecropin, deathin, and defensins in terms of structural modification. Melittin is a short linear sequence with only 26 amino acid residues and no disulfide bond or complex ring structure, which is convenient for artificial solid phase synthesis, truncation, point mutation and multiple types of chemical derivation-modification. In contrast, many insect defensins contain multiple intramolecular disulfide bonds, and their complex spatial structure greatly increases the difficulty of site-directed modification and large-scale preparation [10]. Melittin toxicity and bioactivity can be decoupled through precise site modification: the leucine zipper motif, Trp19, Pro14 and C-terminus cation fragment are all independent functional modules, which can be regulated separately. However, the antibacterial activity and cytotoxicity of most other insect amps are closely coupled and difficult to balance, and the therapeutic index cannot be significantly improved by simple sequence adjustment [11]. Melittin can be combined with chemotherapeutic drugs, gene delivery vectors, tumor-targeting peptides and immunomodulatory sequences to construct peptide–drug conjugates and multifunctional fusion peptides. Melittin can also synergistically reverse bacterial multi-drug resistance with traditional antibiotics, while most other insect amps only exert a single antibacterial effect and lack synergistic compatibility value [12].

Given that no systematic review has yet summarized the research progress on the structural modification of melittin, herein we comprehensively recapitulate its modification strategies and corresponding pharmacological activities to facilitate the further development and application of melittin.

## 2. Sequence-Level Modification

### 2.1. Truncation

Early on, Blondelle (1991) first divided melittin into four functional regions by synthesizing 24 single-residue deletion analogues and analyzing their hemolytic and antibacterial activities: the N-terminal α-helix (1–9), hinge region (10–12), central α-helix (13–20), and C-terminal cationic region (21–26). The study found that the deletion of key residues in the two α-helical regions (e.g., Leu-6, Lys-7, and Trp-19) significantly impaired hemolytic activity, while the deletion of residues in the hinge region or C-terminal region had little effect or slight enhancement, indicating that hemolytic activity is highly correlated with the amphiphilicity of the peptide; in contrast, antibacterial activity is less dependent on sequence changes [14].

Based on this structural understanding, subsequent studies further focused on specific regions. Sun (2005) investigated the role of two Gln residues in the C-terminal fragment Mel (12–26) proposed by Yan, synthesizing a series of truncated and substituted analogs [15]. The results showed that these two Gln residues neither participate in the binding of the peptide to bacterial membranes nor affect the formation of α-helices during binding; their removal instead reduces the entropic cost during the binding process, thereby enhancing antibacterial activity, and the hemolytic toxicity of all analogs is much lower than that of the full-length melittin.

To reduce the high cytotoxicity of melittin at neutral pH and expand its applications, Tan (2012) synthesized the truncated analog MT20 (residues 1–20) [16]. This peptide exhibits reduced membrane lytic activity and cytotoxicity at neutral pH, while retaining high activity at acidic pH. It can effectively promote endosomal escape during gene transfection, significantly improving transfection efficiency without affecting the physicochemical properties of the carrier complex. Therefore, MT20 is considered a safer and more efficient transfection enhancer.

The above study systematically elaborates on the landmark truncation studies of melittin, but has several significant limitations in terms of depth, completeness, and translational evaluation: it relied too much on earlier data on single-residue deletions and fragment truncations, without acknowledging that partial C-terminal truncations, which conflict with later reports, may lose antimicrobial potency against some resistant strains. It interpreted activity changes only in light of amphiphilicity and entropy, without integrating molecular-dynamics simulations to elucidate membrane interactions and atomic mechanisms, and ignored comparative stability. Due to the defects in protease sensitivity and immunogenicity of these truncated peptides in physiological environments, MT20 was considered to be an ideal transfection enhancer solely based on in vitro transfection data, while its potential off-target membrane damage upon increasing dose and the lack of in vivo pharmacokinetic and biosafety validation were ignored.

### 2.2. Point Mutation

In order to overcome the high hemolytic toxicity of melittin and achieve the selective enhancement of its antibacterial activity, the leucine zipper motif in its structure (especially key sites such as Leu-6 and Leu-13) has been identified as a crucial molecular switch, and relevant studies have formed a clear context from mechanism elucidation to rational design. The foundational research by Asthana (2004) first systematically clarified the core role of this motif [17]. The effects of sequence-level modifications on biological properties are summarized in Table 1. By constructing single and double mutants of Leu→Ala, they found that disrupting the leucine zipper reduced hemolytic activity by more than 100-fold while basically retaining antibacterial activity. This confirmed that the motif is the key to regulating hemolytic activity (dependent on α-helix oligomerization and membrane perforation) rather than antibacterial activity, providing a direct target for subsequent selective modification. Based on this target, subsequent studies have developed two sophisticated modification strategies to optimize the biological function of melittin. The first is unnatural amino acid substitution: Radhakrishnan (2024) replaced leucine with its structural isomer 6-aminohexanoic acid (Ahx) and found that substituting only Leu-13 (Mel-LX3) could significantly reduce toxicity to mammalian cells while completely maintaining broad-spectrum antibacterial (including antibiofilm) activity [18]. The second is peptoid modification: Zhu (2007) replaced hydrophobic residues such as leucine with N-substituted glycines (peptoid residues), among which Nphe and Nlys modifications effectively disrupted the peptide’s self-aggregation and membrane perforation capabilities, thereby greatly improving the therapeutic index and shifting the mechanism of action from membrane disruption to potential intracellular targeting [19].

The only tryptophan residue in melittin (Trp19) is the key site for its function and structure. Early studies have systematically elucidated the central role of Trp19. To overcome this technical limitation, subsequent studies have carried out instrumental modifications of this residue. Blondelle (1993) found that Trp19 is the key to membrane insertion and hemolytic activity through full-sequence tryptophan-scanning mutagenesis [20]. Its replacement greatly reduces activity, and the specific position of this residue in the sequence can precisely regulate the strength of the activity. Due to the uniqueness of Trp19 as a core functional site, it has become an important target for subsequent research to understand the membrane-binding mode, conformational change, and structure–function association mechanism of melittin. However, the fluorescence signal of natural tryptophan is easily disturbed by physicochemical factors such as polarity and hydrophobicity of the membrane microenvironment, making it difficult to achieve accurate detection of the dynamic interaction process of this site. In order to overcome this technical limitation, this residue was instrumentally modified in subsequent studies. Lv (2022) replaced Trp19 with the strongly fluorescent unnatural amino acid DapAMCA [21]. This modification not only changed the peptide’s membrane-interaction mode, significantly improved cell selectivity and reduced hemolytic toxicity, but also enabled it to efficiently penetrate cell membranes and specifically localize to the nucleus and nucleolus, thus showing promising prospects as a theranostic molecule. Ridgway (2015) replaced Trp19 with an unnatural analog insensitive to solvent polarity. The resulting analog completely retained the secondary structure, membrane-binding, and biological activity of melittin while obtaining a more stable and reliable fluorescent signal, thereby optimizing its performance as an in situ probe for membrane interactions [22]. Furthermore, researchers have expanded the application potential of melittin through such modifications.

Studies on the rational substitution of key amino acid residues in melittin clearly show a research context: from identifying key sites to precisely regulating functions and then balancing multiple activities. Early studies focused on identifying key residues affecting its basic activity. Rex (2000) was the first to point out that proline P14 in the hinge region is critical for regulating the structural flexibility, self-association, and pore-formation kinetics of melittin [23]. Subsequently, Maher (2010) further systematically studied multiple key sites and confirmed that alanine substitution in the leucine zipper region (L6, L13) can effectively decouple toxicity (hemolysis and cytotoxicity) from intestinal permeability-enhancing activity, while P14→Ala substitution leads to synchronous fluctuations in activity and toxicity due to its complex effects on conformation. These collectively reveal the core role of specific residues in balancing the functions of melittin [24]. With the deepening of research, the focus has shifted to optimizing its selectivity by changing physicochemical properties. A study by Wu (2016) clarified that increasing positive charge is the key to enhancing selective antibacterial activity against Listeria, while simply enhancing hydrophobicity (G1→I) cannot achieve an ideal balance between activity and toxicity [25]. This provides a direction for subsequent functional optimization through precise modification of charged residues.

In recent years, research has entered a new stage of more precise chemical modification of charged residues. Mayandi (2020) replaced α-lysine with ε-lysine, which retained or even enhanced broad-spectrum antibacterial activity while reducing hemolytic and cytotoxicity by approximately 10-fold, achieving a significant improvement in selectivity [26]. The latest study by Matthyssen (2024) further systematically explored the effects of substituting lysine analogs (Orn, Dab, Dap) and found that the charge density and length of side chains (such as Dab, Dap) are key factors for finely regulating antibacterial activity and can simultaneously reduce hemolytic toxicity [27].

### 2.3. D-Amino Acid Conversion

Peptide D-type amino acid modification can significantly enhance the anti-proteolysis ability and prolong the half-life in vivo by steric hindrance effect. The conformation can be fine-tuned to retain/enhance bioactivity and reduce immunogenicity and toxicity. Additionally, some modifications can improve water solubility and membrane permeability. This is particularly valuable for optimizing peptide drugs such as antimicrobial peptides and neuropeptides, providing an effective strategy to maintain both stability and activity [28,29].

As early as 2007, Zhu’s research utilized this strategy to optimize the functional selectivity [30]. They constructed the diastereoisomer Me-D by replacing the key residues in the leucine zipper sequence (Leu-6, Leu-13, Ile-20) with D-type amino acids. This analog maintained or even enhanced the antibacterial activity while reducing hemolytic toxicity to one-eighth of that of the natural melittin, significantly improving cell selectivity. This preliminary study demonstrated the effectiveness of D-type modification in balancing activity and toxicity. Later, they addressed the two core bottlenecks in the clinical application of natural L-melittin: non-specific hemolytic toxicity and the strong anti-vehicle immune response induced by the delivery carrier [31]. They constructed a pH-responsive polymer micelle of full D-type melittin (D-melittin) (DMM) for systemic anti-tumor treatment. It was confirmed that DMM is the first nanocarrier of melittin that can be safely administered systemically, and its safety in vivo is significantly improved compared to natural melittin. However, due to the limitations of the mouse model, formulation science, and insufficient understanding of the immune mechanism, the research is still at the stage of basic animal experiments and needs a lot of targeted optimization, humanized models, and research on druggability to supplement it. The conclusion has a certain degree of uncertainty in human application.

**Table 1:** Summary of sequence-modified melittin analogs and their biological properties.

Name	Author	Modification	Antibacterial/Anticancer Activity	Toxicity	References
MM-2	Asthana	Leu6/Leu13→Ala	-	↓	[17]
Mel-LX3	Radhakrishnan	Leu13→Ahx	-	↓	[18]
ME-l	Zhu	Leu-6/Leu-13/Ile-20→Nleu	↓	↓	[19]
ME-f		Leu-6/Leu-13/Ile-20→Nphe	↓	↓	[19]
ME-k		Leu-6/Leu-13/Ile-20→Nlys	↓	↓	[19]
MELFL	Lv	Trp19→DapAMCA	↑	↓	[21]
AzAla Melittin	Ridgway	Trp19→β1AzAla	-	-	[22]
P14A	Rex	Pro14→Ala	-	-	[23]
PA-1	Maher	Ala Delection	-	↓	[24]
PA-2		Trp Delection	-	↓	[24]
PA-3		Leu6/Leu13→Ala	-	↓	[24]
G1I	Wu	Gly1→Ile	-	-	[25]
I17K		Ile17→Lys	↑	↓	[25]
Mel-4	Mayandi	α-Lys→ε-Lys	↑	↓	[26]
Orn melittin	Matthyssen	Lys7→Orn	-	↓	[27]
Dab melittin		Lys21→Dab	↑	↓	[27]
Dap melittin		Lys23→Dap	↑	↓	[27]
Me-D	Zhu	Leu-6/Leu-13/Ile-20→ D-Amino Acid	↑	↓	[30]
DMM	Lv	D-Amino Acid	↑	↓	[31]

“↑” represents enhanced activity; “↓” represents decreased activity or toxicity; and “-” indicates no significant change or not detected in the study.

## 3. Chemical Modification

### 3.1. Stapling Modification

Stapling modification of peptides can stabilize secondary structures such as α-helices, significantly enhance target affinity and protease resistance, and prolong in vivo half-life. It improves cell-membrane permeability to facilitate targeted intracellular protein–protein interactions and reduces immunogenicity, and some modifications can optimize water solubility. This provides core technical support for peptide drugs to overcome druggability bottlenecks and expand therapeutic applications [32,33].

**Figure 2:**
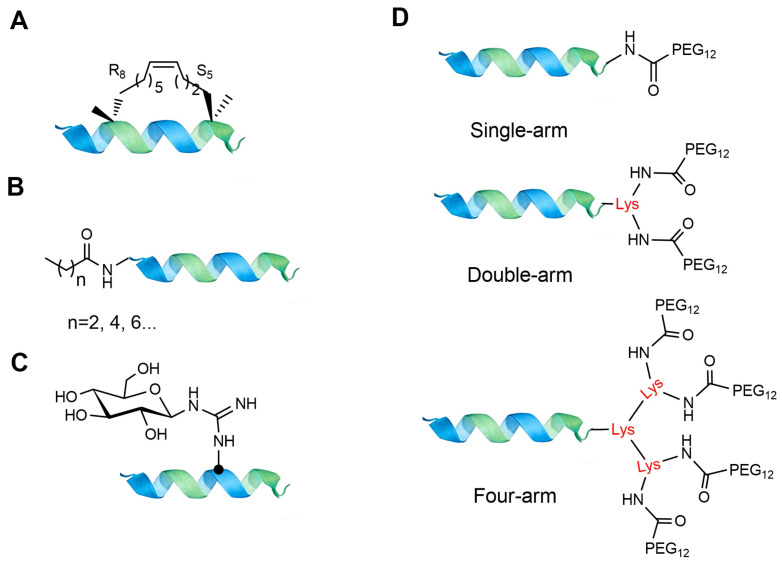
Chemical modifications: (**A**) stapling modification; (**B**) fatty acid modification; (**C**) glycosylation modification; and (**D**) PEG modification. Adapted with permission from References [13,42].

Wu (2017) conducted structural modification of melittin using an all-hydrocarbon stapling strategy (Figure 2A), successfully designing and synthesizing a series of stapled peptide analogs (e.g., Mel-S1 to Mel-S4) and a truncated analog Mel-S5 that form carbon–carbon cross-links at specific spatial positions (i, i + 4 and i, i + 7) [13]. This modification strategy effectively enhanced the α-helical stability of melittin, with the helicity of all stapled peptides (19–47%) being significantly higher than that of native melittin (12%). This structural optimization brought about multiple functional improvements: among them, the analog Mel-S4 exhibited the best anti-hepatocellular carcinoma activity (IC_50_ values of 1.5 μM and 2.0 μM against SMMC-7721 and HepG2 cells, respectively), which was significantly superior to native melittin. Meanwhile, its protease stability was greatly enhanced, with the half-life in α-chymotrypsin prolonged from 4 min (native peptide) to 47 min. In addition, hemolytic toxicity was controlled to a certain extent (HC_50_ = 2.6 μM, better than the native 0.7 μM). This study not only verified the effectiveness of the stapled peptide strategy in optimizing the therapeutic index of melittin but also further identified its key active sites (e.g., Val8, Thr11, Pro14, Leu16) through systematic comparison. It also pointed out that the cross-linking position at the amphiphilic boundary helps improve cell permeability, while the highly polar C-terminal tail is crucial for its activity.

After 2022, new binding chemical systems such as the lysine formidine bond [34] and lactam bridge ring [35], which do not rely on the RU catalyst and do not require exogenous alkene unnatural amino acids, have been developed, solving the inherent defects of traditional RCM binding such as heavy metal residues and hydrophobic bridges that exacerbate hemolysis. However, there are still obvious limitations in the existing studies: most of the binding work only selected a single type of binding bridge for comparison, and lacked parallel control of hydrocarbon RCM, formamidine, and lactam binding under the same cross-linking site, so it is difficult to quantitatively distinguish the independent regulatory effects of different binding frameworks on melittin membrane binding and cell selectivity.

### 3.2. Fatty Acid Modification

Fatty acid modification of peptides can form a “depot effect” through reversible binding with serum albumin, significantly prolonging the in vivo half-life (e.g., GLP-1 analogs can be extended from minutes to days). It enhances protease resistance and membrane permeability, improves pharmacokinetics, increases bioavailability, and reduces immunogenicity, making it suitable for the development of long-acting peptide drugs, especially for the optimization of metabolic and hormonal peptides [36,37].

Native melittin displays poor pharmacokinetic performance. Lacking albumin-binding lipid chains or protective hydrophilic modifications, it is rapidly degraded by circulating proteases, with an ultra-short half-life of only minutes. Its non-specific binding to erythrocytes and somatic tissues accelerates extravascular clearance and lowers plasma exposure, and its high immunogenicity further reduces bioavailability. Similar to the lipophilic alkyl chains of lipopeptides, N-terminal fatty acylation improves the membrane affinity and activity of antimicrobial peptides [38]. Huang (2024) synthesized N-terminally acylated melittin variants with fatty acids of C2–C16 chain lengths (Figure 2B) [39]. Fatty alkyl chains create an albumin-bound circulating depot to slow clearance, while greatly boosting protease stability (over 10-fold for Mel-C8), collectively extending melittin’s half-life from minutes to hours and lowering total clearance. Chain length dictates the overall PK, antibacterial and hemolytic profiles.

These results confirm that fatty acid chain length balances melittin’s antibacterial activity, hemolytic toxicity and protease stability by tuning its amphiphilicity, secondary structure and membrane interactions, alongside key pharmacokinetic indicators including half-life, clearance and tissue distribution.

### 3.3. Glycosylation Modification

Glycosylation of peptides can enhance water solubility and conformational stability, improve resistance to proteolysis through steric hindrance or electrostatic effects, and prolong in vivo half-life. It reduces immunogenicity, optimizes pharmacokinetics, and some sugar chains can confer targeting ability, improving membrane permeability and bioavailability. This is a key strategy for optimizing the druggability of peptide drugs, especially suitable for metabolic peptides such as GLP-1 [40,41].

Yang (2025) conducted systematic N-glycosylation modification of arginine (Arg) residues at positions 22 and 24 of melittin, successfully constructing 24 mono-/di-glycosylated derivatives (Figure 2C) [42]. This strategy achieved synergistic optimization of multiple biological activities of the peptide. The study found that glycosylation first provided a more favorable structural basis for melittin’s membrane interaction, and the α-helicity of some derivatives was significantly improved. At the functional level, the modification effectively decoupled its antibacterial activity from hemolytic toxicity: multi-branched derivatives exhibited better antibacterial activity against specific pathogenic bacteria (e.g., *Streptococcus pneumoniae*) than native melittin, while their hemolytic toxicity was greatly reduced. Among them, the half-maximal hemolytic concentration (HC_50_) of the rhamnose-modified derivative MLT-3c reached 199.3 μM, much higher than that of native melittin. In addition, this modification significantly enhanced the stability of melittin against protease (e.g., trypsin) degradation, improving its in vivo retention capacity as a potential drug. Combining the above advantages, key derivatives (e.g., MLT-3c) achieved the highest therapeutic index among all analogs, indicating that site-specific N-glycosylation at arginine residues is an efficient strategy to simultaneously optimize the activity, selectivity, and stability of melittin.

### 3.4. PEG Modification

PEG modification of peptides can significantly improve water solubility and formulation stability, enhance resistance to proteolysis through steric hindrance effect, and reduce renal clearance to prolong in vivo half-life. It lowers immunogenicity and antigenicity, decreases toxic and side effects, and also improves pharmacokinetic properties and increases bioavailability. This provides a mature and feasible optimization strategy for the long-acting and low-immunogenicity development of peptide drugs [43,44].

Chen (2025) took melittin as a model medicinal peptide and designed and synthesized melittin peptides modified with single-arm, double-arm, and four-arm PEG12 using lysine side chains as branching points (Figure 2D), systematically investigating the regulatory effect of multi-arm PEG conjugation modification on the biological activity spectrum of melittin. The study found that with the increase in the number of PEG arms, the cytotoxicity and hemolytic activity of melittin were significantly reduced (IC_50_ increased by about 20 times), and serum stability was greatly enhanced. The mechanism mainly stems from the steric hindrance effect of PEG, the improvement of peptide water solubility, and the optimization of amphiphilic balance. Moreover, the multi-arm PEGylated derivatives still retained partial antibacterial and antitumor activities while reducing toxicity, ultimately confirming that the multi-arm PEG topology can effectively regulate the activity-toxicity balance of medicinal peptides [45]. In the same year, he added PEG of different lengths to the C and N termini of melittin. The results showed that N-terminal PEGylation significantly reduced the cytotoxicity and hemolytic activity of melittin, while enhancing its proteolytic stability, and these beneficial properties were gradually enhanced with increasing PEG length. In contrast, C-terminal PEGylation has a limited effect in modulating melittin toxicity [46]. The above studies systematically revealed that N-terminal multi-arm PEGylation can significantly reduce toxicity and improve serum stability through steric hindrance and amphiphilic equilibrium optimization, which was ingeniously designed and of paradigm value. However, this work has not yet quantified the therapeutic index gain between “retention of activity” and “reduced toxicity”, nor has it provided direct evidence of peptide conformational changes in the membrane environment. In our view, the “shielding cage” effect of multi-arm PEG is better than that of traditional linear modification, but it is necessary to be careful as excessive PEG may weaken the membrane-cleavage mechanism of melittin which plays antibacterial and anti-tumor roles, and make the “detoxification” become “off-target”. In the future, efforts should be focused on the design of an N-terminal reversible linker and its validation at the in vivo pharmacokinetic and membrane-interaction-simulation level to translate the statistical IC_50_ change into real clinical benefits.

## 4. Conjugation

### 4.1. Peptide–Peptide Conjugation

To overcome the high hemolytic toxicity of melittin and expand its applications, researchers have widely adopted the hybrid peptide strategy. The design logic of this approach has gradually evolved from basic functional fusion to precise targeted delivery and intelligent, responsive release.

Early studies focused on optimizing performance by fusing with other functional peptide segments. For example, Wu (2014) fused the N-terminal hydrophobic fragment of melittin with the core fragment of the antimicrobial peptide LL37, and the resulting hybrid peptide M-L significantly reduced hemolytic toxicity while enhancing antibacterial activity and stability [47]. Different conjugation category and effect on activity and toxicity are summarized in Table 2. Similarly, Jiang (2019) fused α-helical melittin with β-sheet antimicrobial peptide Thanatin, and the constructed hybrid peptide also retained antibacterial activity and reduced toxicity [48]. These studies indicate that rational domain fusion is an effective approach to improve the therapeutic window of melittin. The study by Wu and Jiang confirmed that moderate disruption of the intact helical structure of natural melittin can reduce its non-specific membrane-perforation effect on normal erythrocytes while retaining its bactericidal ability on prokaryotic cell membranes, which is the most direct and low-cost optimization scheme to reduce the toxicity of melittin in the early stage. However, unreasonable fragment fusion can lead to excessive loss of peptide helix structure, which greatly weakens antibacterial activity while reducing hemolytic toxicity. The therapeutic index of some heterozygous peptides is even lower than that of natural melittin. Molecular-dynamics simulation was introduced to quantitatively predict the binding mode of heterozygous peptides to the cell membrane, and the optimal fusion site and linker length were screened in the computer to reduce blind experiments and realize the precise optimization of peptide structure.

To further achieve selective killing, subsequent studies introduced targeting modules. Zhao (2013) conjugated melittin with the hepatocellular carcinoma-targeting peptide AM-2, which significantly enhanced the binding and killing ability of the hybrid peptide to HepG2 hepatocellular carcinoma cells while greatly reducing toxicity to normal hepatocytes, confirming that targeting peptides can guide melittin to enrich specific cells [49]. On the basis of targeting, more sophisticated designs incorporated environmental response mechanisms to achieve controlled release. Sahsuvar (2023) constructed a conjugate consisting of a cervical cancer-targeting peptide, a disulfide bond linker, and melittin [50]. This design enables active melittin to be specifically released only under the high glutathione conditions of the tumor microenvironment, thereby achieving up to 8-fold selective cytotoxicity and effectively inhibiting tumor growth in vivo. This strategy holds prominent potential for specific cell targeting within the tumor microenvironment. Even so, the redox-responsive hybrid peptide designed in this work relies solely on glutathione to trigger melittin activation. Notably, glutathione accumulates at high levels in healthy organs including the liver and kidneys [51]; this would prematurely cleave disulfide linkages in non-tumor tissues and consequently induce undesired off-target toxic effects. At the same time, the tumor microenvironment has multiple abnormal characteristics such as low pH, high matrix metalloproteinases, and high reactive oxygen species [52]. A single stimulation-response mode cannot adapt to the complex physiological environment in vivo, and the accuracy of tumor targeting is still limited.

Lee (2019) used melittin as a targeting carrier and conjugated it with the pro-apoptotic peptide dKLA to construct the hybrid peptide MEL-dKLA, which can specifically induce the apoptosis of M2-like tumor-associated macrophages (TAMs) and effectively inhibit tumor growth [53]. Subsequently, Kim (2022) further optimized this hybrid peptide by truncating the melittin fragment and performing PEG modification, resulting in an upgraded version of PEG-melittin-dKLA (8–26) with higher in vivo stability and complete absence of hemolytic activity, which exhibited stronger anti-tumor and anti-metastatic effects in a breast cancer model [54]. This design expands the application scenario of melittin from the direct killing of bacteria and tumor cells to immune regulation in the tumor microenvironment. Although Kim et al. improved peptide stability by PEGylation, long-term PEG modification would cause accelerated clearance effect in blood, and the enrichment efficiency of peptides at tumor sites decreased significantly after multiple doses [55].

### 4.2. Peptide–Drug Conjugates (PDC)

Peptide–drug conjugates (PDCs) are modular-targeting molecules, which are covalently assembled from three parts: a targeting peptide, a responsive linker and a cytotoxic payload. Peptides specifically recognize highly expressed receptors in tumors to achieve precise delivery of small-molecule drugs. PDC can covalently couple tumor-targeting peptides and cytotoxic drugs through linkers, which has both peptide-targeting recognition and strong killing activity of small-molecule drugs [56].

Due to its efficient membrane-penetration and targeting capabilities, melittin has been extensively explored as a carrier for peptide–drug conjugates (PDCs) to construct intelligent antitumor drug-delivery systems, with related studies demonstrating diverse design strategies and advantages.

To endow melittin with environment-responsive release functionality, Huang (2024) designed an acidic pH-sensitive analog by replacing basic amino acids in melittin with histidine, and on this basis, conjugated the chemotherapeutic drug camptothecin (CPT) to the C-terminus and N-terminus of melittin via disulfide bonds to construct a pH-responsive PDC [57]. The study found that C-terminal conjugation better retained the peptide’s membrane-penetration activity. This conjugate is activated under the acidic conditions of the tumor microenvironment, killing tumor cells through the synergistic effects of drug release and membrane disruption, and exhibits lower toxicity compared to the parent drug and native melittin.

Another strategy emphasizes leveraging its natural cell-targeting ability. Jeong (2023) used melittin as a specific targeting carrier for M2-like tumor-associated macrophages (TAMs) and conjugated it with the potent chemotherapeutic drug maytansine DM1 to construct the PDC drug M-DM1 [58]. In a melanoma model, this conjugate can precisely eliminate tumor-promoting M2-TAMs, effectively inhibit tumor growth, prolong mouse survival, and induce antitumor immune-cell infiltration. Compared to free DM1, M-DM1 significantly enhances efficacy while reducing systemic toxicity, verifying the potential of melittin as a targeted PDC carrier.

The above study supports the application potential of bee-venom peptides as PDC carriers from two aspects: pH microenvironment response and M2-type TAM targeting. However, there are still significant deficiencies. The above study merely highlights the anti-tumor advantages of the conjugates, completely avoiding the recognized transformation bottlenecks such as hemolysis toxicity, in vivo enzyme degradation, immunogenicity, premature disulfide bond cleavage, etc., and did not conduct a chain analysis combining the multiple abnormal characteristics of the liver and kidneys with the tumor microenvironment. In the future, the discussion can be improved by upgrading linker- and peptide-modification techniques, adopting biotopic site-directed coupling processes, supplementing discussions on deficiencies and transformation bottlenecks, and expanding combined treatment and computational simulation studies in five directions to enhance the rigor, cutting-edge nature, and completeness of the content.

**Table 2:** Melittin conjugates and labeled derivatives: linkers, targets and biological properties.

Peptide/Drug	Author	Linker	Bacteria/Cancer	Activity	Toxicity	References
LL37(17–30)	Wu	SUMO	G+/G−	↑	↓	[47]
Thanatin	Jiang	Ala-Gly-Pro	SMMC-7721	↑	↓	[48]
AM-2	Zhao	A(EAAAK)_2_	HepG2	↑	↓	[49]
CSP-KQ	Sahsuvar	-S-S-	HeLa, SiHa	↑	↓	[50]
dKLA	Lee	Gly-Gly	M2-TAM	↑	↓	[53]
dKLA (8–26)	Kim	PEG(N)	M2-TAM	↑	↓	[54]
CPT	Huang	Scheme 1C	B16-F10	↑	↓	[57]
DM1	Jeong	-S-MCC-	SK-MEL-5	-	↓	[58]

“↑” represents enhanced activity; “↓” represents decreased activity or toxicity; and “-” indicates no significant change or not detected in the study.

## 5. Discussion

Compared with natural melittin, each technical route can improve the therapeutic index to varying degrees, but it has its own tradeoff in terms of toxicity reduction, activity retention effect, preparation difficulty and tissue-targeting ability. The main defect of the conjugation system is the cumbersome multi-step synthesis process, high production costs, and the possibility of the linker breaking prematurely in organs rich in glutathione such as the liver and kidneys. From the perspective of the overall efficacy–toxicity balance, multi-stimulus-responsive targeted conjugates have the best performance, providing cell specificity and controllable activation ability for the treatment of lesions, and significantly expanding the therapeutic window for intravenous systemic administration. From the perspective of long-term translational value, multi-layer composite modification integrating sequence mutations, chemical shielding, and targeted conjugation is the most promising direction. It can simultaneously solve multiple issues such as toxicity, metabolic instability, poor selectivity, and pharmacokinetic defects, and will dominate preclinical and early clinical research in the future.

Currently, Apitox produced by the Korean company Apimeds is an approved injectable bee-venom preparation, used to alleviate rheumatoid arthritis, tendinitis, and inflammation-related pain in multiple sclerosis; this product is a bee-venom mixture extract containing bee-venom peptides, bee-venom melittin, phospholipase A_2_, and other components, and is not a purified single-modified bee-venom peptide [59]. The crude bee-venom raw material containing natural bee-venom peptides can be used as a cosmetic additive and does not belong to regulated therapeutic drugs, and there is no unified systemic exposure safety standard [60]. All the structural-modified bee-venom peptide derivatives summarized in this review are at the preclinical research stage, and only in vitro cell experiments and mouse xenograft models have been evaluated. As of 2026, there are no complete or ongoing Phase I–III human clinical trials using synthetic modified bee-venom peptides for systemic anti-tumor, anti-infection, and anti-inflammatory purposes in any of the global mainstream clinical trial databases such as ClinicalTrials.gov in the United States and ChiCTR in China. A few exploratory pilot studies have used crude bee-venom acupuncture for chronic pain, but the trials have limitations such as small sample size, lack of blinding, and non-standard evaluation indicators for efficacy, and the tested drugs are not purified bee-venom peptides. In terms of regulatory submission, there are no public new drug research applications (IND) for modified synthetic bee-venom peptides in the United States, the European Union, and China. Although there are numerous patents related to truncation, book-binding, glycosylation, PEGification, and conjugated bee-venom peptide derivatives, the intellectual property achievements have not been transformed into formal clinical trial application documents. The modified bee-venom peptides have not yet entered any human clinical trials. No single bee-venom peptide polypeptide drug has been submitted as an IND application, nor are there commercialized drugs containing only modified bee-venom peptides. All the evidence of transformation is limited to preclinical animal experiments.

Overall, structural modification fundamentally resolves the contradiction that the efficacy and toxicity of natural bee-venom peptides cannot be balanced. The multifunctional targeted conjugates are the most feasible direction for the long-term development of systemic bee-venom peptide drugs. To promote the clinical transformation of modified bee-venom peptides, this field needs to break away from the simple in vitro and mouse efficacy characterization research mode, and focus on conducting standardized GLP toxicological evaluation, multi-species pharmacokinetic verification, development of low-cost and scalable synthesis processes, and optimization of dual-stimulus-responsive composite modified lead molecules. In the short term, modified bee-venom peptides are only applicable for topical antibacterial use, intratumoral injection, and subcutaneous inflammation and lesion administration, avoiding the risk of systemic hemolysis. Future research can focus on the synergistic composite modification strategy, simultaneously achieving toxicity reduction, improvement of metabolic stability, enhancement of tissue-targeting ability, and designing targeted experiments to narrow the gap between laboratory-ideal results and clinical practical therapeutic value.

## 6. Conclusions

Melittin is a promising natural bioactive peptide restricted by severe hemolytic toxicity, poor in vivo stability and low bioavailability. Three major modification strategies, including sequence modification, chemical modification and conjugate engineering, have been proven effective to rebalance the bioactivity and biosafety of melittin. Truncation, point mutation and D-amino acid substitution precisely regulate peptide structure and cell selectivity. Chemical modifications such as stapling, fatty acylation, glycosylation and PEGylation greatly improve protease resistance, pharmacokinetic properties and half-life. Peptide–peptide and peptide–drug conjugates further realize targeted delivery and stimulus-responsive release.

Current research has clarified the key functional sites and action mechanisms of melittin. Future studies should focus on combined modification strategies, multifunctional intelligent conjugates, large-scale production optimization and systematic preclinical safety evaluation. With further exploration, modified melittin analogs will be transformed into safe and potent therapeutic agents for antibacterial, antitumor and anti-inflammatory applications.

## Data Availability

The data used in this study are obtained from publicly available sources and cited references.
